# Supplementary material for: Omicron Subvariants Infection Kinetics and Nirmatrelvir Efficacy in Transgenic K18-hACE2 Mice
Source: Int J Mol Sci. 2025 Sep 29;26(19):9509. doi: 10.3390/ijms26199509 (PMC12524663; doi:10.3390/ijms26199509)
Supplement: Supplementary file 1 [file ijms-26-09509-s001.zip › ijms-3839398-supplementary.pdf]

Supplementary Material

Omicron Subvariants Infection Kinetics and Nirmatrelvir Efficacy in Transgenic K18-hACE2 Mice.

Vijeta Sharma[1*], Enriko Dolgov[1], Taylor Tillery[1], Camila MendezRomero[1], Alberto Rojas-triana[1], Diana VillalbaGuzman[1], Kira Goldgirsh[1], Risha Rasheed[1], Irene Gonzalez-Jimenez[1], Nadine Alvarez[1], Steven Park[1], Madhuvika Murugan[1], Andrew M. Nelson[1] and David S. Perlin[1*].

[1]Center for Discovery and Innovation, Hackensack Meridian Health, 111 Ideation Way. Nutley, New Jersey 07110, United States.

*Correspondence: (DSP) david.perlin@hmh-cdi.org; (VS) vijeta.sharma@hmh-cdi.org

Supplementary figures:

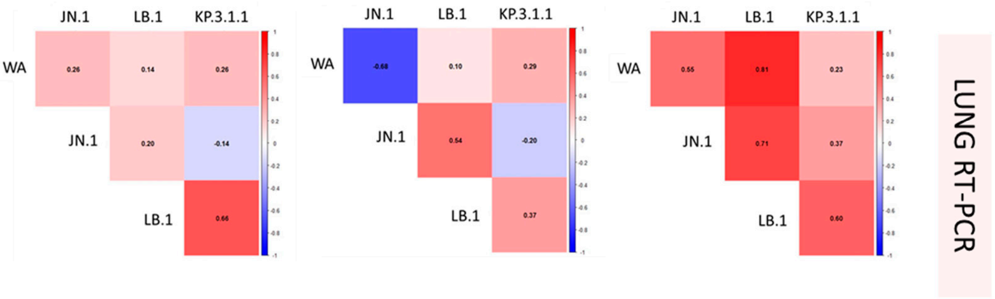

Figure S1. Correlation analysis of the viral RNA among SARS CoV-2 variants at 2, 3, and 4 dpi in K18-hACE2 mice. Spearman correlation matrices calculated in R, showing the relationships between lung viral genomic equivalent levels (Log GE/g) in K18-hACE2 mice infected with SARS-CoV-2 WA1 and Omicron subvariants (JN.1, LB.1, KP.3.1.1) at days 2 (*left*), 3 (*middle*), and 4 (*right*) post-infection. Each cell represents the Spearman's rank correlation coefficient (ϱ) between the viral RNA levels of the variant indicated in the row versus the variant indicated in the column.

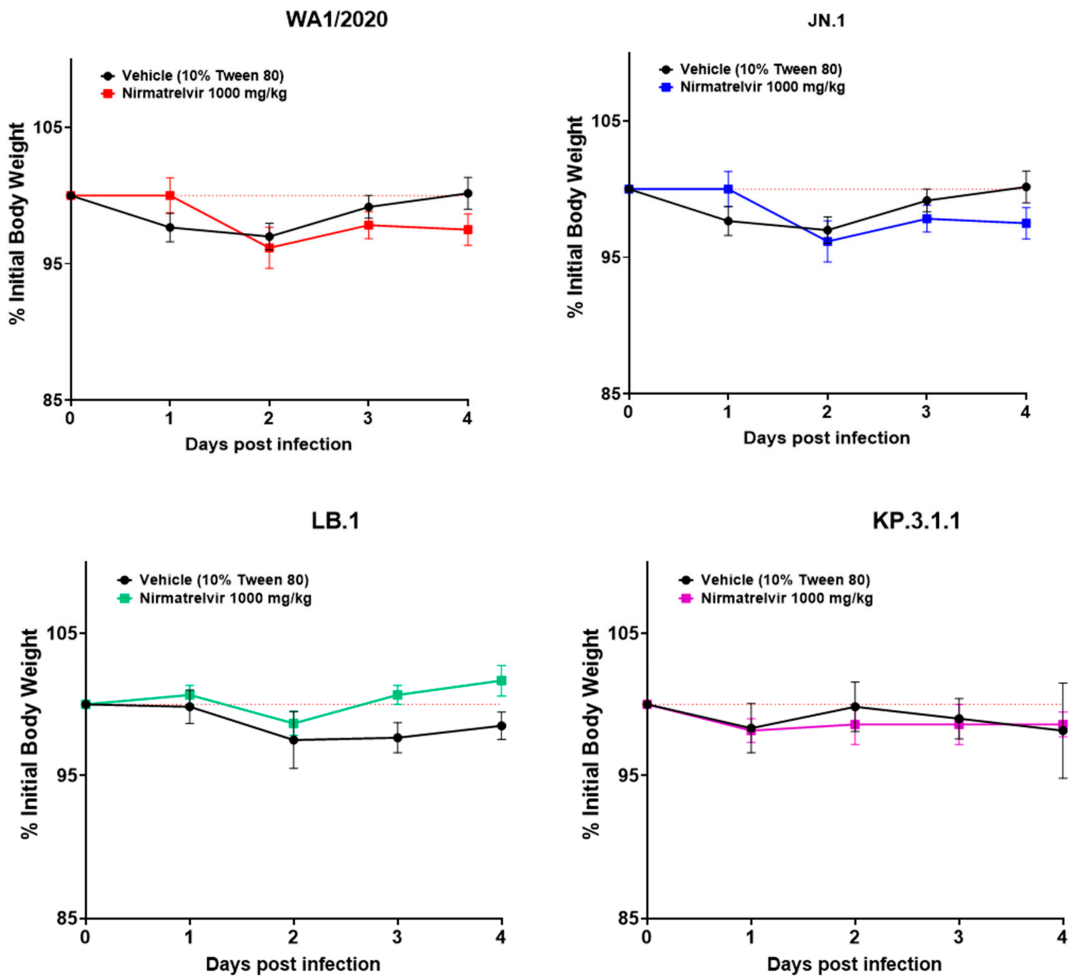

Figure S2. Percent weight change in Nirmatrelvir or vehicle-treated SARS-CoV-2 WA1/2020 or Omicron subvariant-infected K18-hACE2 mice. Line graph showing the percent weight change in Nirmatrelvir or vehicle-treated SARS-CoV-2 WA1/2020 or Omicron subvariant-infected K18-hACE2 mice over the course of treatment post-infection. Percent weight change represented as (% mean ± SEM) in Omicron subvariants JN.1 (*blue*), LB.1 (*green*), KP.3.1.1 (*purple*) and parent WA1/2020 strain (*red*) infected mice.

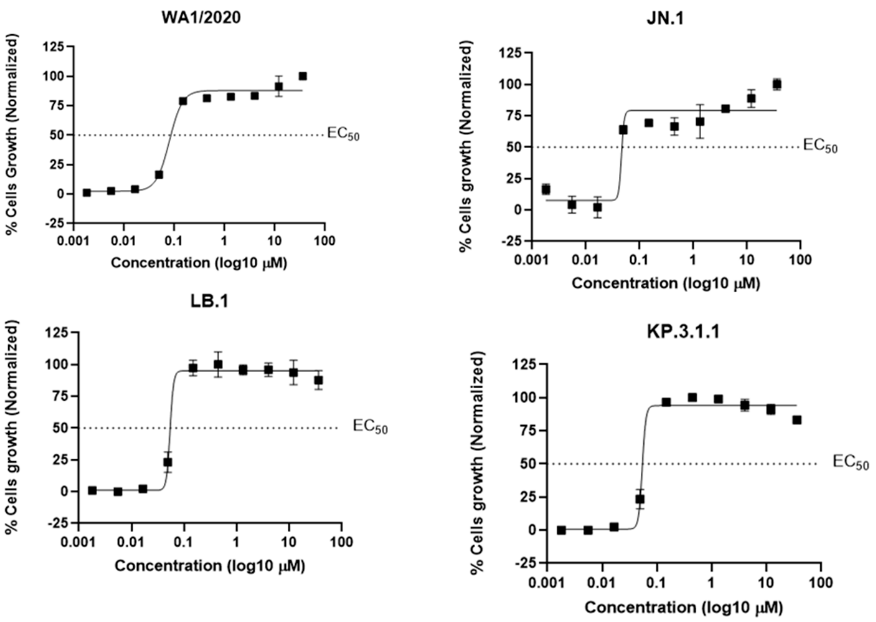

Figure S3. ***In vitro*** **antiviral response Nirmatrelvir against JN.1, LB.1, KP.3.1.1 and WA1/2020**. Dose response semi-log plots showing the half maximal effective concentration (EC_{50}) of Nirmatrelvir's antiviral response against JN.1, LB.1, KP.3.1.1 and WA1/2020. X-axis shows the concentrations (μM) and y-axis shows the percent cell viability normalized to uninfected controls. Data are shown as mean ± s.d.

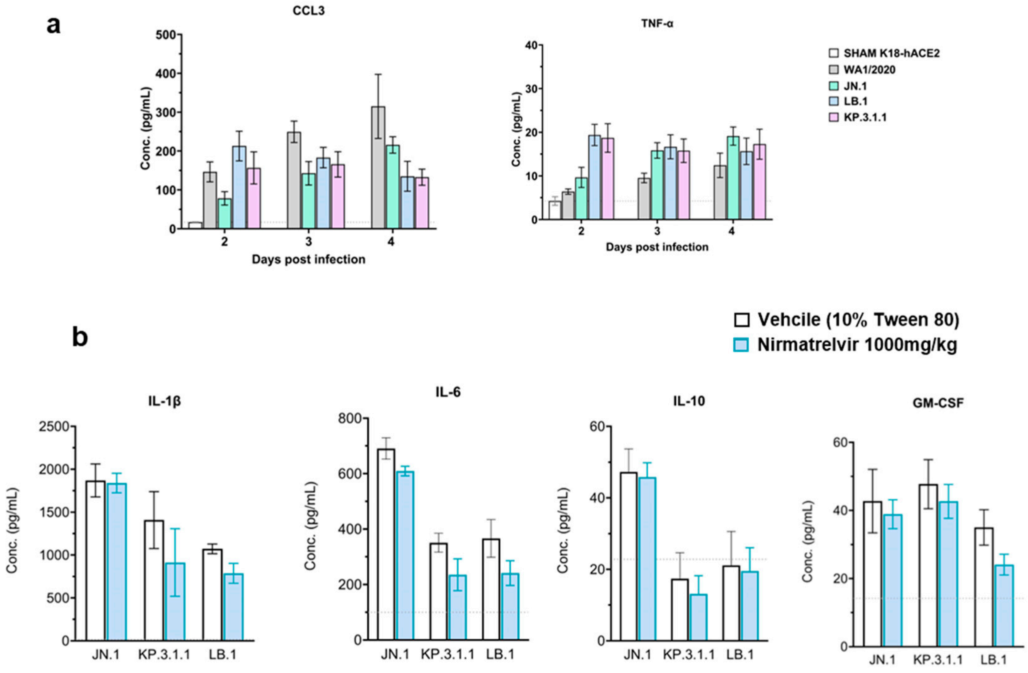

Figure S4. Cytokine and chemokine analysis. **(a)** Bar graphs showing the levels of TNF-α and CCL3 measured in lung homogenates at 2, 3, and 4 dpi in Omicron JN.1, LB.1 and KP3.1.1. infected mice versus WA1/2020. **(b)** Bar graphs showing the levels of IL-10, IL-6, GM-CSF, and IL-1β measured in lung homogenates in Nirmatrelvir (1000 mg/kg) treated JN.1, LB.1 and KP.3.1.1 infected mice versus infected vehicle control. Bar graphs represent concentrations as mean ± SEM pg/mL and dashed lines show baseline levels in uninfected sham control.
